# Supplementary material for: Method for extraction of airborne LiDAR point cloud buildings based on segmentation
Source: PLoS One. 2020 May 29;15(5):e0232778. doi: 10.1371/journal.pone.0232778 (PMC7259685; doi:10.1371/journal.pone.0232778)
Supplement: Attachment — Submitted filename: Plos_one_review_AK.docx [file pone.0232778.s001.docx]

**Manuscript:**	Method for extraction of airborne LiDAR point cloud buildings based on segmentation

| Section | Page | Line # | Comment/suggestion/question |
| --- | --- | --- | --- |
| Abstract | 2 | 21 - 22 | Suggestion: Delete “The airborne ………..to …… ground object. It is also |
|  |  | 22 | Change suggested: The LiDAR technology is an means of ………….. |
|  |  | 35 | Cloud has lower type I …. Instead of, less type I |
|  |  | 35 | Terra Solid, a commercial software. |
| Introduction | 2-4 |  | Introduction needs strengthening, particularly on why this method is needed, and its comparative advantages to currently available open source methods. |
| Data structure and data processing methods | Study area and data |  | Suggestion: Include a section to describe the study area, so as to have a clear view of the test sites and location of these two. |
| Data structure | 5 | 85 | Points in the LiDAR …… instead of The points in the LiDAR |
|  |  | 86 | ….. and unorganized. Suggestion: delete “….. and area unsuitable for …..to ….. interaction. |
|  |  |  | This section needs improvement for clear understanding of the data used. It has missing links. |
|  |  |  | Suggestion: A comparison with other methods, specifically open source would be nice. |
| CSF algorithm | 5 - 6 | 97 | This section needs explanation and clarity, particularly of the principle of the method. |
| PCL-based region growing ….. | 6 | 115 – 117 | Common ……… to region growing segmentation, etc. is an incomplete sentence. Re-write please. |
| Histogram-based building… | 7 | 144 | Suggestion: provide examples and nature of complexity. |
|  |  | 147 | ….in each cluster was obtained after……. |
| Experiment and result analysis | 9  Filter experiment | 166 | Confusing sentence, revise please. The data …… to …. low right. |
|  |  | 170 | 31 points/m2; write and else where as points/m^2^ |
|  |  | 174 | Change: lush to green… surrounded by green vegetation…… |
| Results and analysis |  |  | As the heading of the section is “Experiment and result analysis”, I would suggest to remove this heading here. |
|  |  | 217 | … to be 0…. To be 1….. |
|  |  | 226 | vehicle instead of cars be more appropriate here. |
|  |  | 239 – 240 | Why the thresholds are different between set 1 and set 2. |
|  |  | 242 | Points are calculated as buildings. Instead of mistakenly divided into buildings….. |
|  |  | 249-258 | Reference data used? Or how the errors were calculated and what comparison were made? |
|  |  | 261 | Table 2: Also include total error in addition to Type I and Type II errors. |
|  |  | 270 | Region growth….. |
|  |  | 278 | Is compared with TerraSolid, a commercial software. |
| Figure 1 |  |  | Needs clarity. It doesn’t reflect the phenomena. |
| Figure 2 |  |  | Doesn’t explain … |
| Figure 4 and 5 |  |  | These needs explanation or reflection of building and non-building segments. The color or pattern may be changed. |
